# Isolation of ripening-related genes from ethylene/1-MCP treated papaya through RNA-seq

**DOI:** 10.1186/s12864-017-4072-0

**Published:** 2017-08-31

**Authors:** Yan Hong Shen, Bing Guo Lu, Li Feng, Fei Ying Yang, Jiao Jiao Geng, Ray Ming, Xiao Jing Chen

**Affiliations:** 1 College of Horticulture, Fijian Agriculture and Forestry University, Fuzhou, Fujian 350002 China; 2 Institute of Genetics and Breeding in Horticultural Plants, Fujian Agriculture and Forestry University, Fuzhou, Fujian 350002 China; 3 College of Life Sciences, Fujian Normal University, Fuzhou, Fujian 350117 China; 4 FAFU and UIUC-SIB Joint Center for Genomics and Biotechnology, Fujian Provincial Key Laboratory of Haixia Applied Plant Systems Biology, Fujian Agriculture and Forestry University, Fuzhou, Fujian 350002 China; 5 Department of Plant Biology, University of Illinois at Urbana-Champaign, Urbana, IL61801 USA

**Keywords:** *Carica papaya* L., Firmness, Coloration, Transcriptome, Ethylene, 1-MCP

## Abstract

**Background:**

Since papaya is a typical climacteric fruit, exogenous ethylene (ETH) applications can induce premature and quicker ripening, while 1-methylcyclopropene (1-MCP) slows down the ripening processes. Differential gene expression in ETH or 1-MCP-treated papaya fruits accounts for the ripening processes. To isolate the key ripening-related genes and better understand fruit ripening mechanisms, transcriptomes of ETH or 1-MCP-treated, and non-treated (Control Group, CG) papaya fruits were sequenced using Illumina Hiseq2500.

**Results:**

A total of 18,648 (1-MCP), 19,093 (CG), and 15,321 (ETH) genes were detected, with the genes detected in the ETH-treatment being the least. This suggests that ETH may inhibit the expression of some genes. Based on the differential gene expression (DGE) and the Kyoto Encyclopedia of Genes and Genomes (KEGG) pathway enrichment, 53 fruit ripening-related genes were selected: 20 cell wall-related genes, 18 chlorophyll and carotenoid metabolism-related genes, four proteinases and their inhibitors, six plant hormone signal transduction pathway genes, four transcription factors, and one senescence-associated gene. Reverse transcription quantitative PCR (RT-qPCR) analyses confirmed the results of RNA-seq and verified that the expression pattern of six genes is consistent with the fruit senescence process. Based on the expression profiling of genes in carbohydrate metabolic process, chlorophyll metabolism pathway, and carotenoid metabolism pathway, the mechanism of pulp softening and coloration of papaya was deduced and discussed. We illustrate that papaya fruit softening is a complex process with significant cell wall hydrolases, such as pectinases, cellulases, and hemicellulases involved in the process. Exogenous ethylene accelerates the coloration of papaya changing from green to yellow. This is likely due to the inhibition of chlorophyll biosynthesis and the α-branch of carotenoid metabolism. *Chy-b* may play an important role in the yellow color of papaya fruit.

**Conclusions:**

Comparing the differential gene expression in ETH/1-MCP-treated papaya using RNA-seq is a sound approach to isolate ripening-related genes. The results of this study can improve our understanding of papaya fruit ripening molecular mechanism and reveal candidate fruit ripening-related genes for further research.

**Electronic supplementary material:**

The online version of this article (doi:10.1186/s12864-017-4072-0) contains supplementary material, which is available to authorized users.

## Background

Papaya (*Carica papaya* L.) is one of the most important fruit crops cultivated in tropical and sub-tropical areas and the ripe fruit has a soft and sweet pulp with high amounts of vitamin C, vitamin A, and carotenes. Consumption of fruits rich in vitamin C, carotene, and vitamin E have been associated with a reduced risk of colon cancer. In addition, papaya is a rich source of the digestive enzyme papain, which is used widely in the textile, food, animal feed, chemicals and pharmaceutical industries [1–3]. Global papaya production has grown significantly over the last few years, and papaya is now ranked as the third most popular tropical fruit, behind mango and pineapple [4]. Because papaya is a typical climacteric fruit, it ripens very quickly with striking color changes, substantial pulp softening, and rotting after harvesting. One of the major problems faced by the global papaya industry is significant post-harvest losses throughout the marketing chain. Postharvest losses up to 75% have been reported for papaya fruits shipped from Hawaii to USA mainland [5]. In the Southeast Asia region, post-harvest losses of papaya ranged from 30 to 60%.

Ethylene (ETH) plays a critical role in determining the timing of ripening. Today, supplemental ethylene is commonly used to speed up the ripening of bananas, avocados, mangos in the marketplace. A downside of this treatment is that ethylene shortens the shelf life of many fruits by hastening fruit ripening [6]. Exogenous ethylene can prematurely induce greater endogenous ethylene production, and quicker ripening in climacteric fruit. Exogenous ethylene applications (100 μL·L^−1^) stimulated papaya skin degreening and yellowing, and flesh softening [7]. Researchers have developed several ways to impair the ethylene signaling pathway, including inhibiting ethylene synthesis and perception. Inhibitors of ethylene perception include compounds that have a similar shape to ethylene, but are not able to elicit the ethylene response. A good example of ethylene perception inhibitors is 1-methylcyclopropene (1-MCP). The mechanism of action of 1-MCP involves its tight binding to the ethylene receptor in plants, thereby preventing the binding of ethylene and blocking the effects of ethylene [8]. 1-MCP is used commercially to slow down the ripening of fruits, such as apples, kiwifruits, tomatoes, bananas, plums, persimmons, avocados. 1-MCP can also slow down the ripening of papaya fruit [9, 10]. This is the result of dynamic processes that involve in a complex series of molecular and biochemical changes under genetic regulation.

To better understand the mechanisms of papaya fruit ripening, numerous studies have focused on the analysis of transcript, protein, and metabolite levels in papaya fruits. Using an *Arabidopsis thaliana* microarray, 414 ripening-related genes were identified, and some transcription factors were found in papaya fruit [11]. Twenty-seven protein spots showing differences in abundance during papaya ripening were successfully identified using the 2-DE analysis [9]. Although some studies on papaya ripening have been conducted, little is known about the genetic control of ripening due to technical limitations. RNA-seq is a good method to examine the total RNA levels in different samples. Exogenous ethylene stimulates papaya ripening, while 1-MCP inhibits the ripening progression. Differential gene expression induced by the different treatments accounts for these observations. Therefore, screening for differentially expressed genes will be helpful for further elucidating the fruit ripening molecular mechanism. This study will analyze transcript levels in papaya fruits after 1-MCP or ethylene treatment to isolate the key ripening-related candidate genes, as well attempting to enhance our understanding of papaya fruit ripening molecular mechanism.

## Results

### RNA-seq

**Fig. 1:**
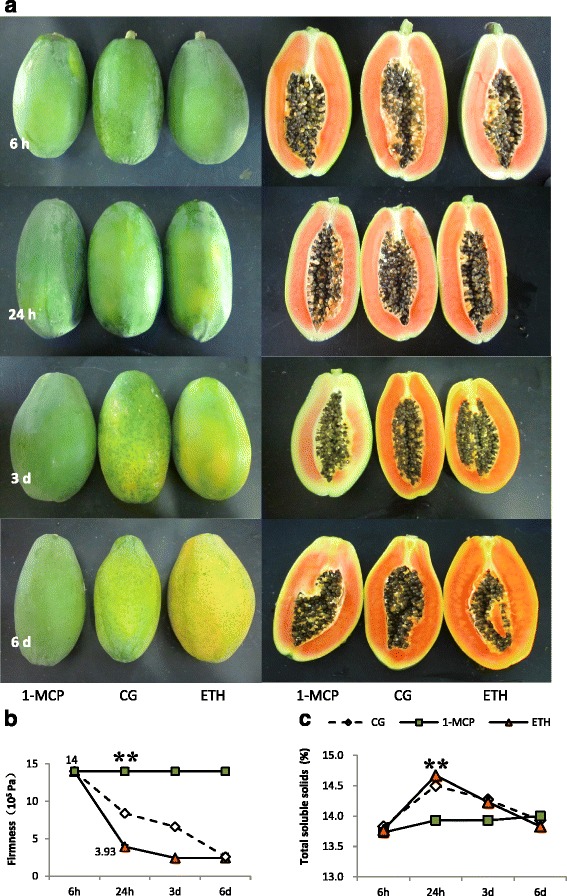
Firmness and total soluble solids of papaya fruits. **a**, Papaya fruits used in the experiment (6 h, 24 h, 3 d, 6 d are the time points after treatments; ETH, 1-MCP, CG represent ETH-treated, 1-MCP-treated or non-treated papaya fruits respectively.); **b**, Firmness; **c**, Total soluble solids. **means significant difference at *P* ≤ 0.01 level

To identify candidate ripening-related genes in papaya, we conducted a large-scale transcriptome analysis of ETH-treated, 1-MCP-treated and untreated papaya fruit using an RNA-seq based approach. Exogenous ethylene accelerates climacteric fruit ripening, whereas 1-MCP slows it down. The effects of exogenous ethylene and 1-MCP treatments on skin color, total soluble solids (TSS), and pulp texture are shown in Fig. 1. In 1 day the pulp firmness of ETH-treated papaya changed from 14 to 3.93 10^5^ Pa, whereas 1-MCP-treated fruits retained pulp firmness at high levels for the entire duration of the experiment (Fig. 1). 1-MCP also delayed papaya fruit skin coloration, and fruits treated with 1-MCP were still green 6 days after treatment (Fig. 1a). The TSS changing trend of 1-MCP-treated papaya was significantly different compared to that of the control and ETH-treatments. The TSS was highest at 24 h and then decreased in control and ETH-treatments, while TSS kept a low level and increased a little in 6 d in 1-MCP-treatments. Papaya fruits treated with 1-MCP showed a significant delay in the softening, color changing, and the formation of soluble solids, In contrast the ETH-treated papayas showed quick softening and color changing, with the differences in firmness and TSS at 24 h among three treatments reaching significant levels (Fig. 1b, c). Therefore, three samples collected at 24 h after treatments were used for RNA extraction, cDNA libraries construction, and sequencing.

**Fig. 2:**
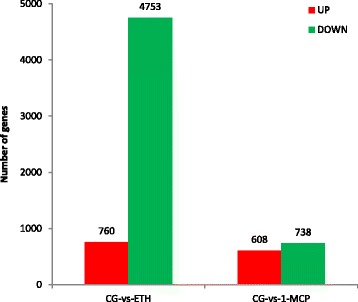
Differential gene expression statistics. The number of up-regulated and down-regulated genes between CG-vs-ETH and CG-vs-1-MCP are summarized. Compared with the untreated papaya (CG), there are 760 genes up-regulated and 4753 genes down-regulated in ETH-treatment; while there are only 608 genes up-regulated and 738 genes down-regulated in 1-MCP-treatment

The main results of RNA-seq are showed in Table 1 and Fig. 2. Table 1 shows the clean reads number and the gene number of the three samples. After removing low-quality reads, adaptor sequences, and rRNA reads, we obtained 43,873,036 (1-MCP), 65,149,940 (CG), and 33,805,002 (ETH) clean reads. Clean reads were then mapped to the papaya reference genome. There were 18,648 (1-MCP), 19,093 (CG), and 15,321 (ETH) genes (including 1127 (1-MCP), 1138 (CG), 1009 (ETH) new genes) were detected, which the genes were detected in the ETH-treatment was the least. The data showed that ETH inhibited the expression of some genes. Compared with the untreated papaya, 760 genes were up-regulated and 4753 genes were down-regulated in ETH-treatment; in the 1-MCP-treated papaya, there were only 608 genes up-regulated and 738 genes down-regulated (Fig. 2). This demonstrates that ETH inhibited more genes than 1-MCP. This may because ETH is an important hormone in fruit ripening, it can affect a lot of genes’ expression.

**Table 1:** The gene number of all samples

Sample Name	Total Clean Reads No.	Known Gene No. (Known Gene Ratio)	New Gene No.	All Gene No.	All Reference Gene No.
1-MCP	43,873,036	17,521 (63.09%)	1127	18,648	27,770
CG	65,149,940	17,955 (64.66%)	1138	19,093	
ETH	33,805,002	14,312 (51.54%)	1009	15,321	

The Known Gene Ratio = Known Gene No. / All Reference Gene No

### DEGs function annotation

Papaya genes were annotated using the Gene Ontology database and genes were classed into three categories (Additional file 1: Figure S1). Among the three categories, the terms of cellular progress and metabolic progress were observed to occur most frequently in the ontology of biological process; while the terms of cell and cell part were observed to occur most frequently in the ontology of cellular component; and the terms of binding, catalytic activity were observed to occur most frequently in the ontology of molecular function. In comparison with the control treatment, the terms of cell killing, reproductive process, reproduction, multi-organism process, extracellular region, and nutrient reservoir activity were up-regulated with ETH-treatment, while the terms of extracellular region, and nutrient reservoir activity were down-regulated with 1-MCP-treatment. This implied that ethylene may accelerate the reproductive process, the induction of death, and nutritious substrate storage. On the contrary, 1-MCP may inhibit the expression of genes in the extracellular region, or genes relating to nutritious substrate storage.

**Fig. 3:**
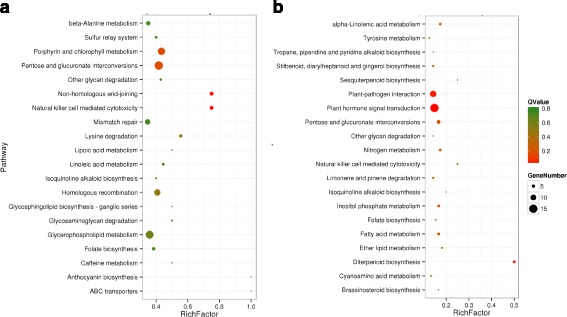
Top 20 pathways in KEGG enrichment by Qvalue. **a**, CG-vs-ETH; **b**, CG-vs-1-MCP. Rich Factor is the ratio of the differentially expressed number of genes located in the pathway and the total number of genes located in the pathway. The higher the Rich Factor, the higher the degree of enrichment. QValue is the *P*-value after the multiple hypothesis test correction, in the range of 0 to 1, the closer to zero, the more significant the enrichment

In addition, the pathway enrichment analysis was carried out using the Kyoto Encyclopedia of Genes and Genomes (KEGG) pathway database. The results showed that DEGs participating in the pathways of pentose and glucuronate interconversions and the porphyrin and chlorophyll metabolism were significantly enriched in the ETH-treated fruits; while DEGs that participated in the pathways of plant-pathogen interaction, plant hormone signal transduction, and diterpenoid biosynthesis, pentose and glucuronate interconversions were significantly enriched in the 1-MCP-treated fruits (Fig. 3). Genes involved in the pentose and glucuronate interconversions pathway are shown in Additional file 2: Figure S2. The 3.2.1.15 in the red frame refers to polygalacturonase (PG), which is the key gene in the pentose and glucuronate interconversions pathway. This gene encodes for a very important enzyme that participates in papaya softening [12]. PG was up-regulated significantly response to ETH treatment and down-regulated after 1-MCP treatment.

### Ripening-related genes selection

**Fig. 4:**
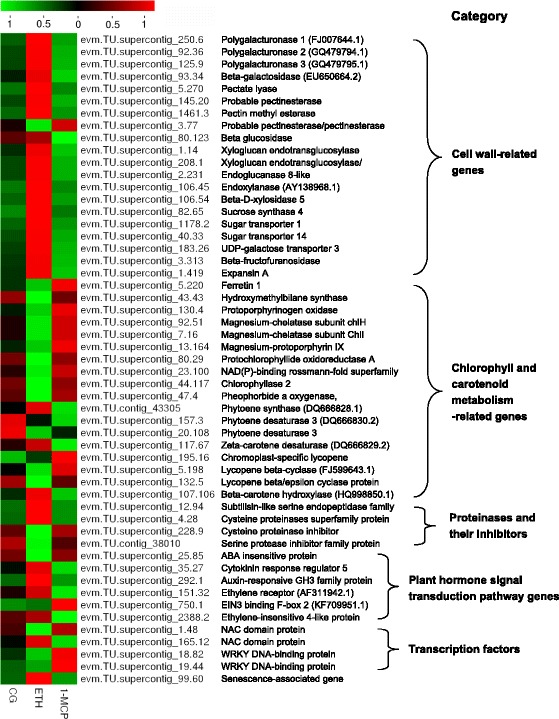
Heat map diagram of expression levels for selected ripening-related genes analyzed by KEGG. The heat map were drawn according to FPKM values. Columns and rows in the heat map represent samples and genes, respectively. Sample names are displayed below the heat map. Color scale indicates fold changes of gene expression

According to the DEGs expression and the KEGG pathway enrichment, 53 genes were selected: 20 cell wall-related genes, 18 chlorophyll and carotenoid metabolism-related genes, four proteinases and their inhibitors, six plant hormone signal transduction pathway genes, four transcription factors, and one senescence-associated gene (Additional files 3: Table S1 and Fig. 4).

Among the cell wall-related genes, except pectinesterase/pectinesterase inhibitor (PMIS), the expression patterns of polygalacturonase (PG), beta-galactosidase (GAL-B), pectate lyase (PL), pectin methylesterase (PME), beta-glucosidase (GLU-B), xyloglucan endotransglucosylase (XTH), endoglucanase 8-like (EGase), endoxylanase (EXY1), beta-D-xylosidase 5 (XYL), sucrose synthase 4 (SUS4), sugar transporter (STP), UDP-galactose transporter 3 (UTR3), beta-fructofuranosidase (BFF), and expansin A (EXPA) were the same. Expression of these genes in the ETH-treatment was higher than that in the control treatment, and lowest in the 1-MCP treatment. PG, GAL-B, PL, and PME are involved in the degradation of pectin. GLU-B, XTH, EGase, EXY1, XYL may be involved in the degradation of cellulose and semi-cellulose. This suggested that ETH induced the expression of PG, GAL-B, PL, PME, GLU-B, XTH, EGase, EXY1, and XYL genes, then accelerated the degradation of pectin, cellulose, and hemi-cellulose, and therefore ethylene treatment results in the rapid softening of papaya fruit. Conversely, 1-MCP inhibited the expression of these genes and the fruits retained pulp firmness. These genes may play important roles in papaya cell wall degradation and fruit softening. PMIS is active against plant PMEs, PMIS showed a very low expression level in ETH-treated papaya. ETH induced the expression of SUS4, STP, UTR3, and BFF genes, promoted the synthesis of sucrose and fructose, and the transport of sugar. Expansins are non-enzymatic proteins found in the plant cell wall, with important roles in plant cell growth, fruit softening, and meristem function. In our study, an expansin A gene showed lower expression level in 1-MCP-treated papaya than that in ETH-treatment and control.

The color of the papaya fruit is determined largely by the presence of chlorophyll and carotenoid [13]. Ethylene applications affect papaya fruit coloration causing it to change from green to yellow. Fig. 3 showed that DEGs involved in the pathways of the porphyrin and chlorophyll metabolism were enriched in the ETH-treatment, Additional file 5: Figure S3 and Additional file 3: Table S1 showed that ETH inhibited chlorophyll metabolism. The major chlorophyll biosynthesis enzymes, including ferretin 1 (FER1), hydroxymethylbilane synthase (HEMC), protoporphyrinogen oxidase (HEMG), magnesium-chelatase subunit chlH (ABAR), magnesium-chelatase subunit ChlI (CH-42), magnesium-protoporphyrin IX methyltransferase (CHLM), and protochlorophyllide oxidoreductase (PORA) were highly inhibited in ETH-treated samples, where chlorophyll biosynthesis was inhibited. Chlorophyll degradation enzymes such as NAD (P)-binding rossmann-fold superfamily protein (NYC1), chlorophyllase 2 (CLH2), pheophorbide a oxygenase, chloroplastic-like (ACD1) were also inhibited. This is indicative that the synthesis and degradation of chlorophyll both decreased in ETH-treated papaya. On the contrary, the expression of ferretin 1 in 1-MCP-treated papaya fruits was about two fold higher than that found in untreated papaya (Additional file 3: Table S1).

In the carotenoid biosynthesis pathway, lycopene beta/epsilon cyclase protein isoform 2 (LCY-B/E) and β-carotene hydroxylase (CHY-B) were enriched in KEGG pathway enrichment (Additional file 4: Figure S4). ETH treatment clearly up-regulated the expression of β-carotene hydroxylase gene, and decreased the expression of *LCY-B/E* (Additional file 3: Table S1). Other key carotenoid synthesis genes including phytoene synthase (PSY), phytoene desaturase 3 (PDS), zeta-carotene desaturase (ZDS), chromoplast-specific lycopene β-cyclase (CYC-B), lycopene beta-cyclase (LCY-B) were also detected, but were not significantly differentially expressed among treatments.

Protein degradation is one of the most significant features of senescence, and a lot of genes up-regulated during senescence are proteases [14]. Several lines of research suggest that serine proteases are strongly associated with senescence in wheat [15, 16], and barley [17]. Cysteine proteases (CPs) are the most abundant class of up-regulated proteases during natural or induced senescence [14, 18]. In our study, we identified one subtilisin-like serine endopeptidase family protein and one cysteine proteinases superfamily protein, which were up-regulated in response to ETH-treatment and down-regulated in 1-MCP-treated papaya. In addition, the expression of one serine protease inhibitor and one cysteine proteinase inhibitor were greatly inhibited by ETH (Additional file 3: Table S1).

KEGG pathway enrichment analysis of the DEGs indicated that genes encoding the plant hormone signaling components were significantly enriched in the 1-MCP-treated samples. Plant hormones regulate a wide range of processes, including the development and ripening of fruit. Ethylene is an important hormone controlling papaya fruit ripening. In ethylene signal transduction pathway, expression of the ethylene receptor ethylene-insensitive 4-like protein (EIN4) was inhibited by 1-MCP, and the expression of EIN3 binding F-box 2(EBF2) increased in response to 1-MCP-treatment. In *Arabidopsis*, overexpression of *F-box* genes display ethylene insensitivity [19]. The high expression levels of *EBF2* in the 1-MCP-treatment may reduce the sensitivity to ethylene. Genes in ABA, cytokinin, and auxin signal transduction pathway, with different expression levels in three treatments were also found.

We also selected several transcription factors and one senescence-associated gene through gene different expression. One NAC domain protein and two WRKY DNA-binding proteins showed an obvious decrease, the other NAC and senescence-associated gene increased in expression with application of ethylene.

### Confirming genes expression using RT-qPCR

**Fig. 5:**
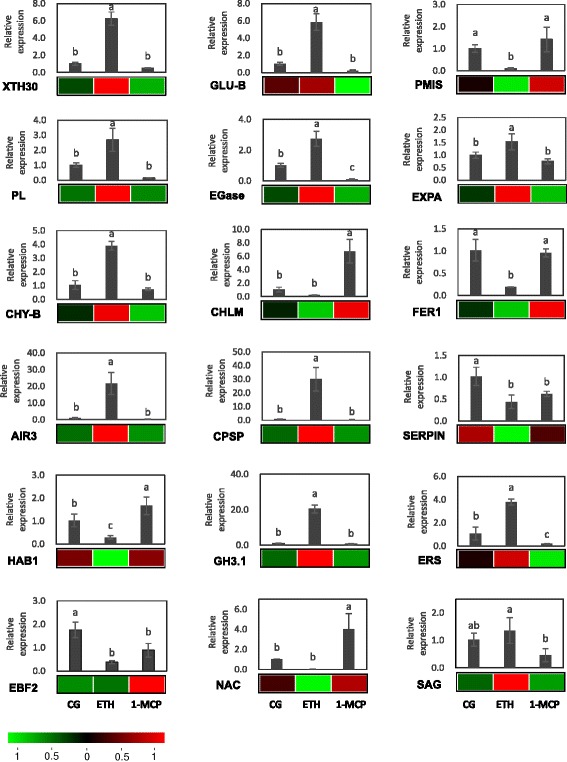
Candidate gene expression levels revealed by RT-qPCR and RNA-seq. The heat map shows FPKM values for the 18 selected candidate genes. Sample names are displayed below the heat maps. Color scale indicates fold changes of gene expression. The bar charts show the RT-qPCR results. RT-qPCR was performed with primer sets in Table 2. *CpEIF* was used as the reference gene. Error bars on each column indicate SDs from three replicates. Different lower case letters above bars indicate statistically significant differences at *P* < 0.05 (one-way ANOVA, Duncan’s tests). Different letters indicate significant differences between groups, while same letters represent no significant difference. The genes and the gene_id are as follows: XTH30 (evm.TU.supercontig_208.1), GLU-B (evm.TU.supercontig_80.123), PMIS (evm.TU.supercontig_3.77), PL (evm.TU.supercontig_5.270), EGase (evm.TU.supercontig_2.231), EXPA (evm.TU.supercontig_1.419), CHY-B (evm.TU.supercontig_107.106), CHLM (evm.TU.supercontig_13.164), FER1 (evm.TU.supercontig_5.220), AIR3 (evm.TU.supercontig_12.94), CPSP (evm.TU.supercontig_4.28), SERPIN (evm.TU.contig_38010), HAB1 (evm.TU.supercontig_25.85), GH3.1 (evm.TU.supercontig_292.1), ERS (evm.TU.supercontig_151.32), EBF2 (evm.TU.supercontig_750.1), NAC (evm.TU.supercontig_1.48), SAG (evm.TU.supercontig_99.60)

To confirm the accuracy and reproducibility of the transcriptome analysis results, 18 genes were selected for RT-qPCR validation. RNA samples from the 24 h ETH-treated papaya fruit were used as templates. Primers of the candidate genes are shown in Table 2. Except *FER1* (evm.TU.supercontig_5.220) and *EBF2* (evm.TU.supercontig_750.1), the expression profiles of the other 16 candidate genes determining using RT-qPCR data were consistent with the RNA-seq results (Fig. 5), thus confirming our transcriptome analysis.

**Table 2:** PCR primers used in this study

Primer	Sequence (5´–3´)
EIF-F	AGGCAGGCAAGAGAAGAT
EIF-R	TTCATACCGAGTAGCGATTC
XTH30-F	AGCGTCCGTCTCCTCCTC
XTH30-R	TCGTCGTGTGTCTTCTCAAATAC
GLU-B-F	ACTGGATGCTAAGTGGTATGAAC
GLU-B-R	GATGTCTCTCTTGGGATTCTTGG
PMIS-F	CACCACCTCCGCCATCTC
PMIS-R	CGCCCTGAACTCCACTTCC
PL-F	CTCTAACTCCCATTTCACCAAACAC
PL-R	ACCTACACCTCGGCATCCTC
EGase-F	CTAAGCCACGCCAATAAGGTT
EGase-R	GGATGAGCCGCAACTGATG
EXPA-F	CGTGAAGGTGAGCGTTAAGG
EXPA-R	TGACCGAACTGCCAATTAGC
CHY-B-F	CTCTCCGCCGCCATTACC
CHY-B -R	TCCTCCAACACGAAGCAGAC
CHLM-F	GAACCACCCTCGTCGTCTC
CHLM-R	TCCTTCACCACTTCCTTGTCC
FER1-F	AGATGGGAACAATGACC
FER1-R	TTACCGACCAACCTCA
AIR3-F	GAAGAAGCATCAGAACTCGTCAAG
AIR3-R	GAGAGAGCCAGAGGGAATTATACC
CPSP-F	GGGAGAGAAGGGTTACATTAGGATG
CPSP-R	TAGGATAGAGTGGAGGTTCAGTAGG
SERPIN-F	CGGTGCCTACGATGGTTTCAAAGTT
SERPIN-R	GAACCCAGAACCAGATCCTAACTTC
HAB1-F	CGAAGGCGGATTCTACTGTG
HAB1-R	GAAGAGCAAGCATTGAGAGGTAA
GH3.1-F	CGAGTGTTACTTTGGCTTGAATCT
GH3.1-R	TGGGCGAATTGGGTGAGT
ERS-F	GGAGGTCACATTTGGATA
ERS-R	TGGTTTATGCCTGGTTAG
EBF2-F	ATCAGCAGCAGCACAGTAGA
EBF2-R	GCCAATACCTGGAAGGGACATA
NAC-F	TCAGTCTCGTAAGCAATCCAA
NAC-R	TGTGGTTGTGGCATTTCTTCC
SAG-F	TGTGGTTCTCATACGGTCTGCT
SAG-R	CAAAGGTTCCGGCAGCTTCTTG

### Transcription levels of six selected genes during different processing

**Fig. 6:**
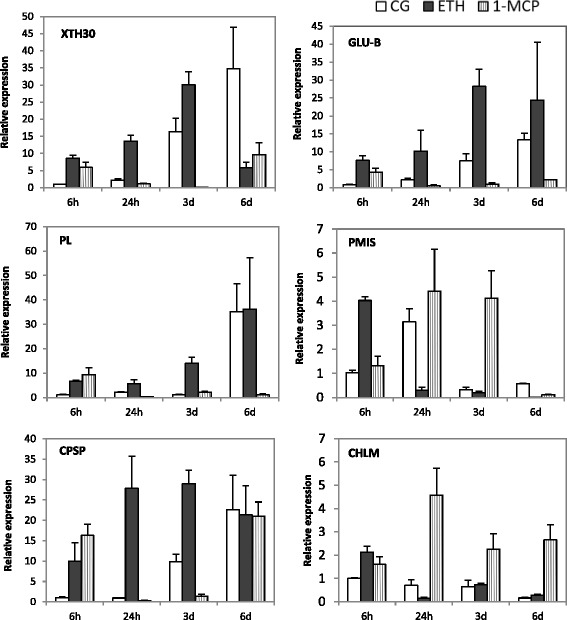
Transcription levels of six selected genes during different processing. Transcript levels of six selected genes during different processing were measured through RT-qPCR. RT-qPCR was performed with primer sets in Table 2. *CpEIF* was used as the reference gene. Error bars on each column indicate SDs from three replicates. 6 h, 24 h, 3 d, 6 d are the time points after treatments

Six fruit ripening-related candidate genes were selected and their transcription levels in ETH-treated, 1-MCP-treated, and non-treated papaya fruits were analyzed by RT-qPCR (Fig. 6). RT-qPCR analysis revealed that the expression patterns of *XTH*, *GLU-B*, and *PL* were similar. The expression of these genes was highly induced by ethylene and inhibited by 1-MCP. In addition, the expression pattern is consistent with the fruit senescence process and fruit softening, indicating that the three genes are involved in fruit softening in papaya. PMIS inhibits the expression of PMEs, which are known to catalyze the demethoxylation of partial purification and characterization of pectinmethylesterase [20]. Thus, during fruit ripening, PME may play an important role in determining the extent of pectin degradation. *PMIS* showed very low expression levels in ETH-treated papaya, while *PMIS* retained a high level in 1-MCP-treatment in 24 h and 3 d. This indicated that high levels of expression of *PMIS* inhibited the expression of PME in 1-MCP-treated papaya, thus preventing pectin degradation. The expression of one proteinase gene was also analyzed. *CPSP* showed extremely up-regulated and a high expression level maintained in ETH-treatment. The expression of *CPSP* increased during the process of fruit ripening and reached the highest level at 6d in 1-MCP-treated and control papayas. CHLM is one of the key chlorophyll biosynthesis enzymes, the expression of *CHLM* was maintained at a lower level in ETH-treated and control papayas than the 1-MCP-treatment.

## Discussion

To help in elucidating the molecular basis of ripening process in papaya, 24 h ETH-treated, 1-MCP-treated and untreated papaya fruits were analyzed by RNA-seq. 18,648 (1-MCP), 19,093 (CG), and 15,321 (ETH) genes were detected, with thousands of genes showing differential expression. According to the KEGG enrichment analysis and differential gene expression analysis, about 50 ripening-related genes were isolated. Because fruit color and firmness are the major fruit quality parameters, we focused on the analysis of genes related to fruit softening and coloration.

Pulp softening renders the fruit better for eating but harder for storage and transport. Firmness is one of the most important fruit quality features characterizing fruit quality. Papaya is a soft fruit with a short postharvest shelf life. The primary cell wall of fruits is a structure mainly composed of polysaccharides (pectin, cellulose, and hemi-cellulose). During papaya fruits ripening, pectin, cellulose, and hemi-cellulose depolymerisation increase [21, 22]; endo- and exo-polygalacturonase (PG), pectin methyl esterase (PME), glucanase, galactosidase, and xylanase have been detected in ripening papaya fruits [23–25]. In our study, several cell wall degrading candidate genes were selected, such as the genes of polygalacturonase, beta-galactosidase, pectin methyl esterase, pectate lyase, xyloglucan endotransglucosylase, endoxylanase, endoglucanase 8-like. Our results are consistent with the previous research.

Among the cell wall degrading candidate genes, PGs, GAL-B, PME, PL are important pectinolytic enzymes which are responsible for the solubilisation of pectins during papaya ripening. PGs and GAL-B play a central role in pectin solubilization during papaya fruit ripening [26, 27]. Three PG genes were detected, the expression patterns were same but the expression abundance among the three genes were different. The fpkm of *Pg1* in ETH-treatment is 15,642.8, while that of *Pg2* and *Pg3* are only 12.09 and 32.85. There were more than 10 beta-galactosidase genes detected, only *Gal-b* (evm.TU.supercontig_93.34) was abundantly expressed in ethylene-treated papaya and was inhibited by 1-MCP, with the expression pattern being consistent with fruit softening [12]. Because more abundant gene transcripts account for more enzymatic activity participating in softening, *Pg1*(evm.TU.supercontig_250.6) and *Gal-b* (evm.TU.supercontig_93.34) are the major cell wall degrading genes involved in the solubilization of pectins.

Several genes that could be involved in the degradation of cellulose and semi-cellulose were also selected, including *Glu-b*, *Xth*, *Egase*, *Exy1*, and *Xyl*. Endoglucanase and beta-glucosidase are key enzymatic component involved in cellulose hydrolysis [28]. *Exy1*, *Xyl* [29], and *Xth* [30] are important genes participating in the degradation of hemicelluloses, thus playing an important role in plant cell wall remodeling. Some of the genes participating in fruit softening in other species have been identified, including the beta-glucosidase gene in sweet cherry [31], *FaXyl1* in strawberry [29], *Ma-Xth2* and *Ma-Xth10* in apple, *Ad-Xth*4,5 and 7 in kiwifruit [30].

Cell wall disassembly during fruit ripening is a cooperative process involving the coordinated expression of multiple genes. The expression of sucrose synthase 4, sugar transporter, beta-fructofuranosidase genes were highly up-regulated in ETH-treated papaya, which had the highest TSS and lowest firmness. This suggested that ETH improves the rate of sugar synthesis, transport and degradation. We also found that the transcripts of the expansin A gene were abundant in the ETH-treatment sample. Although expansin is non-enzymatic protein, it is well known to play important roles in fruit softening [32, 33]. The papaya genome contains at least 15 expansin A genes [34], only the expression of expansin A (evm.TU.supercontig_1.419) was associated with firmness changes, indicating that this gene may be involved in the papaya fruit softening.

To confirm our hypotheses, the expression of *Xth30*, *Glu-b*, and *Pl* genes was measured in ETH-, 1-MCP-, and Non-treated papaya fruits by RT-qPCR. We found that the expression of three genes was ripening-related and correlated with the variation in softening patterns of different treatments. Therefore, we suggested that the high expression levels of *Xth30*, *Glu-b*, and *Pl* genes may be related to the degradation of the papaya fruit cell wall. In general, besides the cell wall-related genes that have been cloned (e.g. *Pg*, *Gal-b*, *Pl*, *Xth32*, and *Exy1*), several novel genes (e.g. *Pme*, *Pmis*, *Glu-b*, *Egase*, *Xyl*, *Expa*) were selected for further analysis. We illustrate that papaya fruit softening is a complex process with significant cell wall hydrolases, such as pectinases, cellulases, and hemicellulases participating in the process. Among all the cell wall-related genes, *Pg1* was strongly induced by ETH and was highly inhibited by 1-MCP, and the expression abundance was very high. Therefore, *Pg1* may play the most important role in the fruit softening of papaya.

Exogenous ethylene applications accelerate the climacteric fruit ripening process, with the most visible change being the color of exocarp turning from green to yellow during papaya fruit ripening. Papaya fruits treated with 1 μL·L^−1^ 1-MCP retained the green color 6 days post treatment. The color of the papaya fruit is determined largely by the presence of chlorophyll and carotenoids [13]. Typically, young fruits are green, due to the high contents of chlorophyll in young fruits. Then a steady increase in carotenoids and simultaneous rapid loss of chlorophyll causes a color shift from green to yellow during fruit ripening [35, 36]. We analyzed the genes involved in chlorophyll and carotenoid metabolism. ETH highly inhibited the expression of several chlorophyll biosynthesis enzyme genes such as *Fer1*, *Hemc*, *Hemg*, *Abar*, *Ch-42*, *Chlm*, and *Pora*, therefore inhibiting chlorophyll biosynthesis.

‘Da Qing No 7’ is a red-fleshed papaya. The pulp of full-ripe fruit is red and the peel is yellow. β-cryptoxanthin, lycopene, and β-carotene are the major carotenoids in these red-fleshed papaya fruits [37, 38]. Subsequent red-fleshed fruit maturation leads to a gradual accumulation of β-cryptoxanthin and total lycopene [39]. Thus, we speculated that the accumulation of β-cryptoxanthin may be the main reason for the yellow peel, β-carotene hydroxylase (*Chy-b*) was enriched in the KEGG pathway, and the expression of *Chy-b* was highly inhibited by 1-MCP and boosted by ETH. β-carotene hydroxylase is a key enzyme in the pathway of carotenoid biosynthesis in plants, which catalyses the conversion of β-carotene to β-cryptoxanthin [40, 41]. 1-MCP inhibited the expression of *Chy-b*, while ETH increased the expression of this gene, illustrating that CHY-B is a key enzyme in β-cryptoxanthin synthesis and may be responsible for the yellow color of papaya fruit.

The other key carotenoid synthesis genes including *Psy*, *Pds*, *Zds*, and *Cyc* were also found to be expressed in all treatments. There were no significant differences in the expression of *Psy*, *Pds*, *Zds*, and *Cyc* among the three treatments. *Lcy-b/e* was enriched in the KEGG pathway. Cyclization of lycopene is a key branch point in the carotenoid biosynthetic pathway, the biosynthetic pathway of carotenoids splits at the level of lycopene into the α-branch and the β-branch [42]. As we can see from Additional file 6 Figure S5, in α-branch, lycopene ε-cyclase catalyzes the cyclization of lycopene to form α-carotene; In β-branch, lycopene β-cyclase catalyzes the cyclization of lycopene to form β-carotene [43]. The expression of *Lcy-b/e* in ETH-treated papaya has a significant lower transcriptional level than that in untreated papaya, the expression of the lycopene ε-cyclase genes were down-regulated by ethylene in orange too [44], this suggests that ethylene may inhibit the α-branch and β-branch may be the main synthetic pathway in the later stage of fruit ripening [37]. Besides *Lcy-b/e* and *Lcy-b*, chromoplast-specific lycopene beta-cyclase (*Cyc-b*) was detected as well. A 2-bp insertion is present in the coding region of the recessive red flesh allele resulting in a frame-shift mutation and a premature stop codon, so as to accumulate a large amount of lycopene which is responsible for the red flesh [45]. In ‘Da Qing No 7’, lycopene beta-cyclase (LCY-B, evm.TU.supercontig_5.198) is in β-branch and may be responsible for the biosynthesis of β-carotene, which are then converted to β-cryptoxanthin, thus contributing to the yellow peel of papaya. The expression pattern of *Chy-b* is consistent with the β-cryptoxanthin accumulation. Thus, we speculated that the accumulation of β-cryptoxanthin may be responsible for the yellow color of papaya fruit.

## Conclusions

Fruit ripening is a complex, developmentally regulated process requiring the participation of numerous genes. Exogenous ethephon (0.5 g·L^−1^) treatment stimulated papaya ripening, while 1-MCP (1 μL·L^−1^) treatment inhibited ripening progression and made the fruits retain a “rubbery” texture [46]. Differential gene expression induced by the different treatments accounts for these observations. Therefore, comparing the differential gene expression in ETH-treated and 1-MCP-treated papaya using RNA-seq is a sound approach to isolate papaya ripening-related genes. Numerous cell wall-related genes, color-related genes, proteinases, plant hormone signal transduction pathway genes, transcription factors, and a senescence-associated gene were isolated in our study. Among these genes, several genes had already been isolated and characterized as players in papaya ripening, corroborating our findings. These include the *Pg* and *Gal-b*, that play a central role in pectin solubilization during papaya fruit ripening [26, 27]. RT-qPCR were used to analyze the transcription levels of several selected genes under different treatments. The results also confirmed the RNA-seq data and verified that the expression pattern of the six analyzed genes is consistent with the fruit senescence process. These findings strongly suggest that these genes may be involved in the ripening of papaya fruit. Nevertheless, the specific role of these genes in fruit ripening remains unclear, and further work is required to accurately characterize the function of these genes in fruit ripening. The mechanisms of pulp softening and coloration of papaya were also deduced and discussed in the paper. We illustrate that papaya fruit softening is a complex process with significant cell wall hydrolases, such as pectinases, cellulases, and hemicellulases involved in the process. *Pg1* may play the most important role in the fruit softening of papaya. Exogenous ethylene accelerates the coloration of papaya changing from green to yellow. This is likely due to the inhibition of chlorophyll biosynthesis and the α-branch of carotenoid metabolism. *Chy-b* may play an important role in the yellow color of papaya fruit. Our results deepen our understanding of the molecular mechanisms regulating papaya fruit ripening, and provide candidate fruit ripening-related genes for further research.

## Methods

### Plant materials and RNA preparation

Papaya fruits (*C. papaya* L. cv. ‘Daqing No.7’) at the mature-green stage were harvested from a local commercial plantation in Zhangzhou, China. The healthy fruits of similar size, shape, and maturity were separated into three groups and treated at 25 °C. Thirty-six fruits were incubated with 1 μL·L^−1^ of 1-methylcyclopropene (1-MCP) gas for 18 h in a sealed box; 36 fruits were dipped into 0.5 g·L^−1^ of ethephon solution for 3 min, then dried and put in a sealed box for 2 h; the 36 control fruits (Control Group, CG) were dipped into water for 3 min, then dried and put in a sealed box for 2 h, with three replicates. After treatments, all fruits were stored at 25 °C and allowed to ripen. Fruits were taken randomly at 6 h, 24 h, 3 d, and 6 d after treatments. The firmness of papaya was measured with a GY-3 firmness meter and the total soluble solids (TSS) were measured with a pocket refractometer. Three biological replicates were taken. Three fruits were peeled, seeds were removed, and the flesh was cut into some pieces. The pieces of papaya pulp were mixed, frozen in liquid nitrogen, and stored in - 80 °C. Total RNA was extracted from the papaya pulp using an RNA extraction kit (DongSheng Biological Technology Ltd., Guangzhou, P. R. China).

### Sequencing, assembly and gene annotation

Total RNA of three samples (CG 24 h, ETH 24 h, 1-MCP 24 h) was used to prepare cDNA libraries using the Illumina Dynabeads® mRNA DIRECT™ Kit. Then, the cDNA libraries were used for paired-end 125 sequencing using an Illumina Hiseq2500 at Genedenovo Biotechnology Co., Ltd. (Guangzhou, China). In total, three sets of raw reads were obtained, and all sequencing data were deposited in the NCBI Sequence Read Archive (SRA).

The raw reads were filtered to remove “dirty” data, including low-quality reads, adaptor sequences, and rRNA reads, to generate “clean” reads. The estimate of gene expression and identification of differentially expressed genes (DEGs) were conducted using a modified method described previously [47]. FPKM (Fragments Per Kilobase of transcript per Million mapped reads) was used to measure the transcript abundance of each gene, those with a fold-change of ≥2 and a false discovery rate (FDR) < 0.05 were considered significant DEGs [48]. All of the genes were annotated using the reference papaya genome [49] database (*Carica papaya* Version1.181:CpGDB181(JGI)), NCBI non-redundant (Nr) database, the Kyoto Encyclopedia of Genes and Genomes (KEGG) pathway database. The KEGG enrichment analysis were performed with a Qvalue cut off of 0.05.

### RT-qPCR analysis

For RT-qPCR, oligonucleotide primers were designed according to each gene’s 3′-untranslated region with DNAMAN (Table 2). *CpEIF* was used as the reference gene [50]. RT-qPCR was carried out using SYBR Green-based PCR assay in a Bio-Rad CFX96 Real-Time PCR System (Bio-Rad, USA). Each reaction mix contained 1.0 μL of cDNAs, SYBR Premix ExTaq™ 10 μL, PCR forward primer (10 μmol·L^−1^) 0.5 μL, PCR reverse primer (10 μmol·L^−1^) 0.5 μL, ddH_2_O 8.0 μL, to a final volume of 20 μL. The PCR conditions were 95 °C for 3 min, followed by 40 cycles of 95 °C for 15 s, 56 °C for 30s, and 72 °C for 35 s. Each RT-qPCR analysis was performed in triplicate, and the mean was used for RT-qPCR analysis. The relative expression of the genes was calculated according to the method of 2^−△△Ct^ [51], and SPSS was used to analyze the data.

## Additional files

Additional file 1: Figure S1. Gene Ontology classification of genes. A, CG-vs-ETH; B, CG-vs-1-MCP. (DOCX 558 kb)

Additional file 2: Figure S2. KEGG graph of pentose and glucuronate interconversions pathway (only part of the pictures were shown). A, CG-vs-ETH; B, CG-vs-1-MCP. Genes with a red frame are up-regulated DEGs, while down-regulated genes are inside a green frame; genes inside a half red half green frame belong to gene families containing both up- and down-regulated DEGs. The 3.2.1.15 in the frame refers to polygalacturonase (evm.TU.supercontig_250.6, evm.TU.supercontig_92.36). (DOCX 58 kb)

Additional file 3: Table S1. Selected ripening-related genes. (DOCX 23 kb)

Additional file 4: Figure S3. KEGG graph of porphyrin and chlorophyll metabolism pathway (CG-vs-ETH). 1.16.3.1 indicates ferretin 1 (evm.TU.supercontig_5.220); 2.5.1.61 indicates hydroxymethylbilane synthase (evm.TU.supercontig_43.43); 1.3.3.4 indicates protoporphyrinogen oxidase (evm.TU.supercontig_130.4); 6.6.1.1 indicates magnesium-chelatase subunit ChlI (evm.TU.supercontig_7.16) and magnesium-chelatase subunit chlH (evm.TU.supercontig_92.51); 2.1.1.11 indicates magnesium-protoporphyrin IX methyltransferase (evm.TU.supercontig_13.164); 1.3.1.33 indicates protochlorophyllide oxidoreductase A (evm.TU.supercontig_80.29); 1.1.1294 indicates NAD (P)-binding rossmann-fold superfamily protein (evm.TU.supercontig_23.100); 3.1.1.14 indicates chlorophyllase 2 (evm.TU.supercontig_44.117); 1.14.1220 indicates pheophorbide a oxygenase, chloroplastic-like (evm.TU.supercontig_47.4). (DOCX 85 kb)

Additional file 5: Figure S4. KEGG graph of carotenoid biosynthesis pathway. A, CG-vs-ETH; B, CG-vs-1-MCP. The CruA indicates lycopene beta/epsilon cyclase protein (evm.TU.supercontig_132.5); The CrtR and CrtZ indicate beta-carotene hydroxylase (evm.TU.supercontig_107.106). (DOCX 57 kb)

Additional file 6: Figure S5. General pathway of carotenoid metabolism. (DOCX 26 kb)

## Abbreviations

1-MCP: 1-methylcyclopropene
ABAR: Magnesium-chelatase subunit chlH
ACD1: Pheophorbide a oxygenase, chloroplastic-like
BFF: Beta-fructofuranosidase
CG: Control Group
CH-42: Magnesium-chelatase subunit ChlI
CHLM: Magnesium-protoporphyrin IX methyltransferase
CHY-B: β-carotene hydroxylase
CLH2: Chlorophyllase 2
CYC-B: Chromoplast-specific lycopene β-cyclase
DGEs: Differentially expressed genes
DGE: Differential gene expression
EGase: Endoglucanase 8-like
ETH: Ethylene
EXPA: Expansin A
EXY1: Endoxylanase
FER1: ferretin 1
FPKM: Fragments Per Kilobase of transcript per Million mapped reads
GAL-B: Beta-galactosidase
GLU-B: Beta-glucosidase
HEMC: Hydroxymethylbilane synthase
HEMG: Protoporphyrinogen oxidase
KEGG: Kyoto Encyclopedia of Genes and Genomes
LCY-B: Lycopene beta-cyclase
LCY-B/E: Lycopene beta/epsilon cyclase protein isoform 2
NYC: NAD (P)-binding rossmann-fold superfamily protein
PDS: Phytoene desaturase
PG: Polygalacturonase
PL: Pectate lyase
PME: Pectin methylesterase
PMIS: Pectinesterase/pectinesterase inhibitor
PORA: Protochlorophyllide oxidoreductase A
PSY: Phytoene synthase
RT-qPCR: Reverse transcription quantitative PCR
STP: Sugar transporter
SUS4: Sucrose synthase 4
TSS: Total soluble solids
UTR3: UDP-galactose transporter 3
XTH: Xyloglucan endotransglucosylase
XYL: Beta-D-xylosidase 5
ZDS: Zeta-carotene desaturase
