# Supplementary figures and images for: Isolation of ripening-related genes from ethylene/1-MCP treated papaya through RNA-seq
Source: BMC Genomics. 2017 Aug 31;18:671. doi: 10.1186/s12864-017-4072-0 (PMC5580268; doi:10.1186/s12864-017-4072-0)

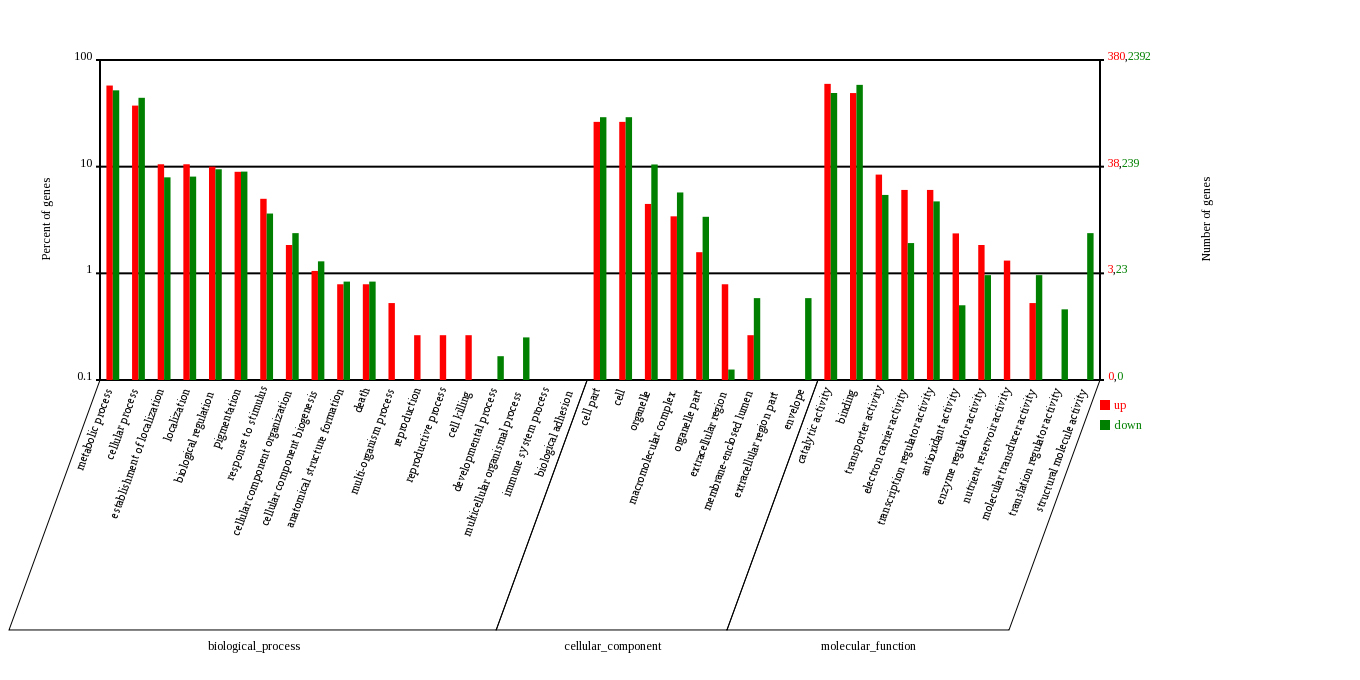


**A**


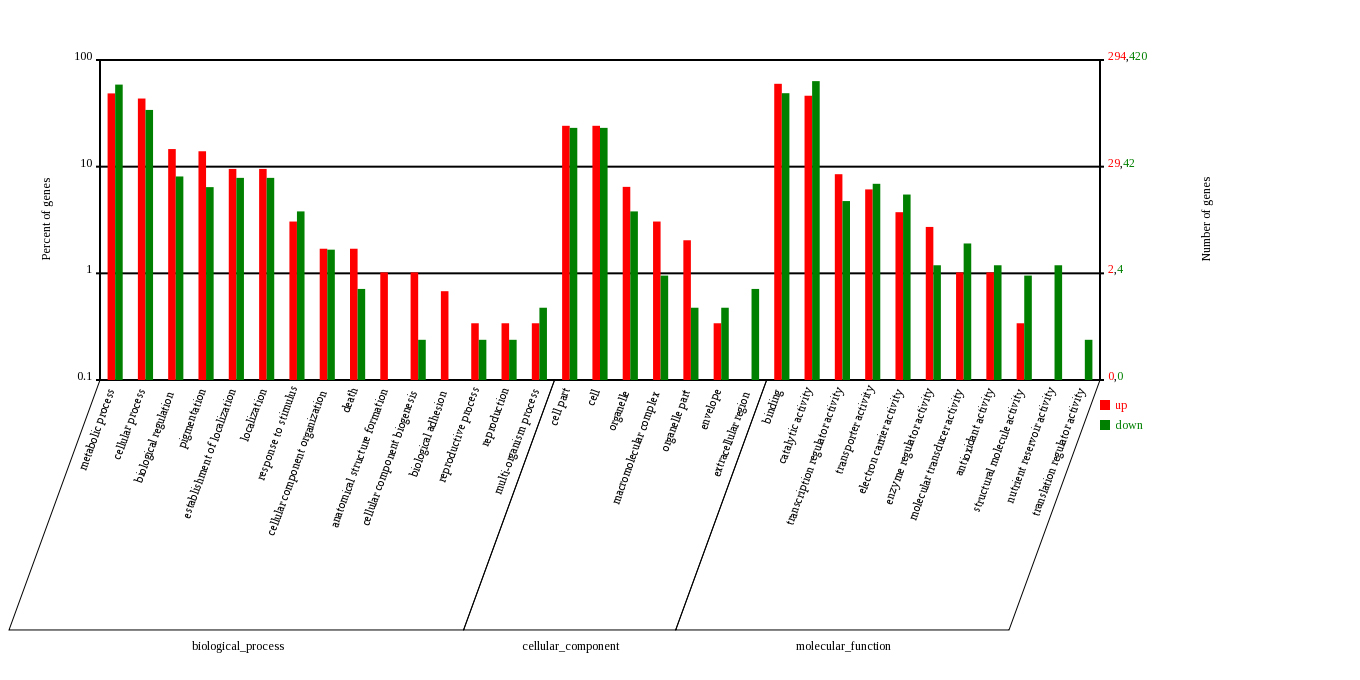


**B**

**Additional figure 1 Gene Ontology classification of genes**

A, CG-vs-ETH; B, CG-vs-1-MCP.

Supplement: Supplementary file 1 — Gene Ontology classification of genes. A, CG-vs-ETH; B, CG-vs-1-MCP. (DOCX 558 kb) [file 12864_2017_4072_MOESM1_ESM.docx]
