# Supplementary material for: Isolation of ripening-related genes from ethylene/1-MCP treated papaya through RNA-seq
Source: BMC Genomics. 2017 Aug 31;18:671. doi: 10.1186/s12864-017-4072-0 (PMC5580268; doi:10.1186/s12864-017-4072-0)
Supplement: Supplementary file 2 — KEGG graph of pentose and glucuronate interconversions pathway (only part of the pictures were shown). A, CG-vs-ETH; B, CG-vs-1-MCP. Genes with a red frame are up-regulated DEGs, while down-regulated genes are inside a green frame; genes inside a half red half green frame belong to gene families containing both up- and down-regulated DEGs. The 3.2.1.15 in the frame refers to polygalacturonase (evm.TU.supercontig_250.6, evm.TU.supercontig_92.36). (DOCX 58 kb) [file 12864_2017_4072_MOESM2_ESM.docx]

**A**

**
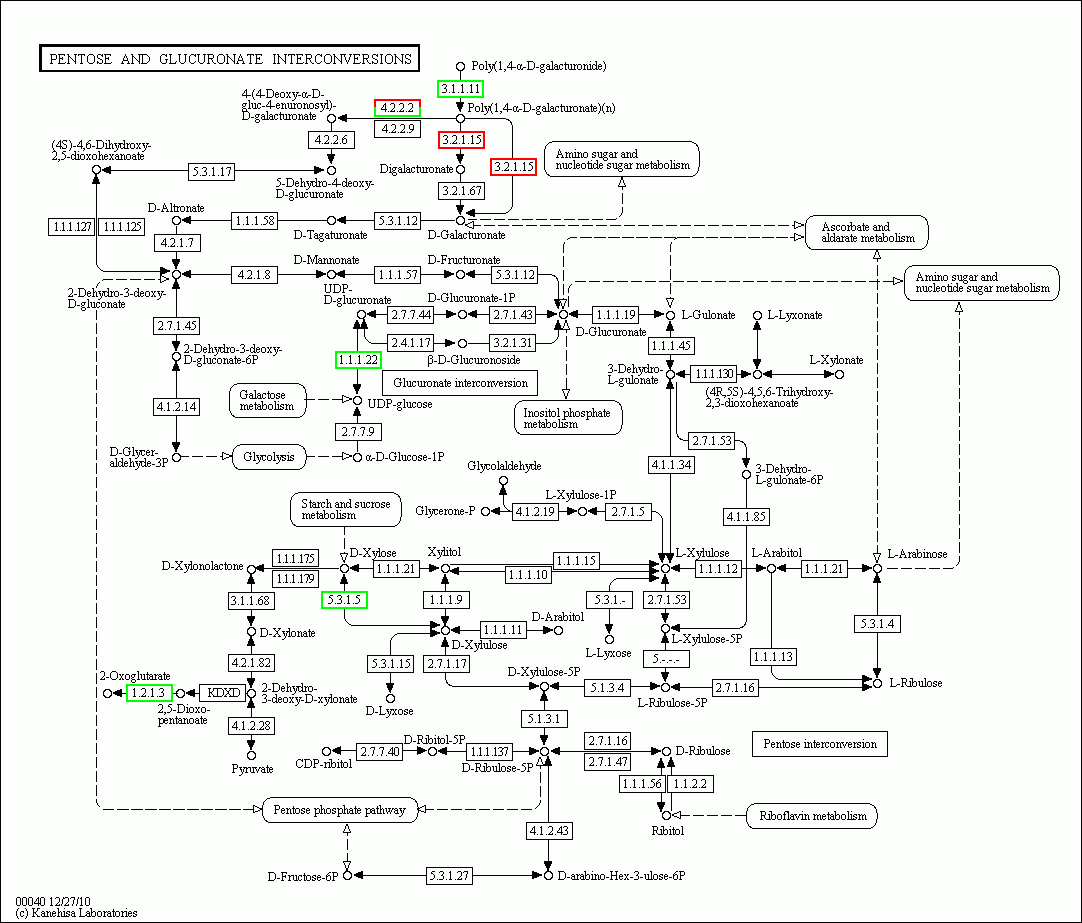
**

**B**


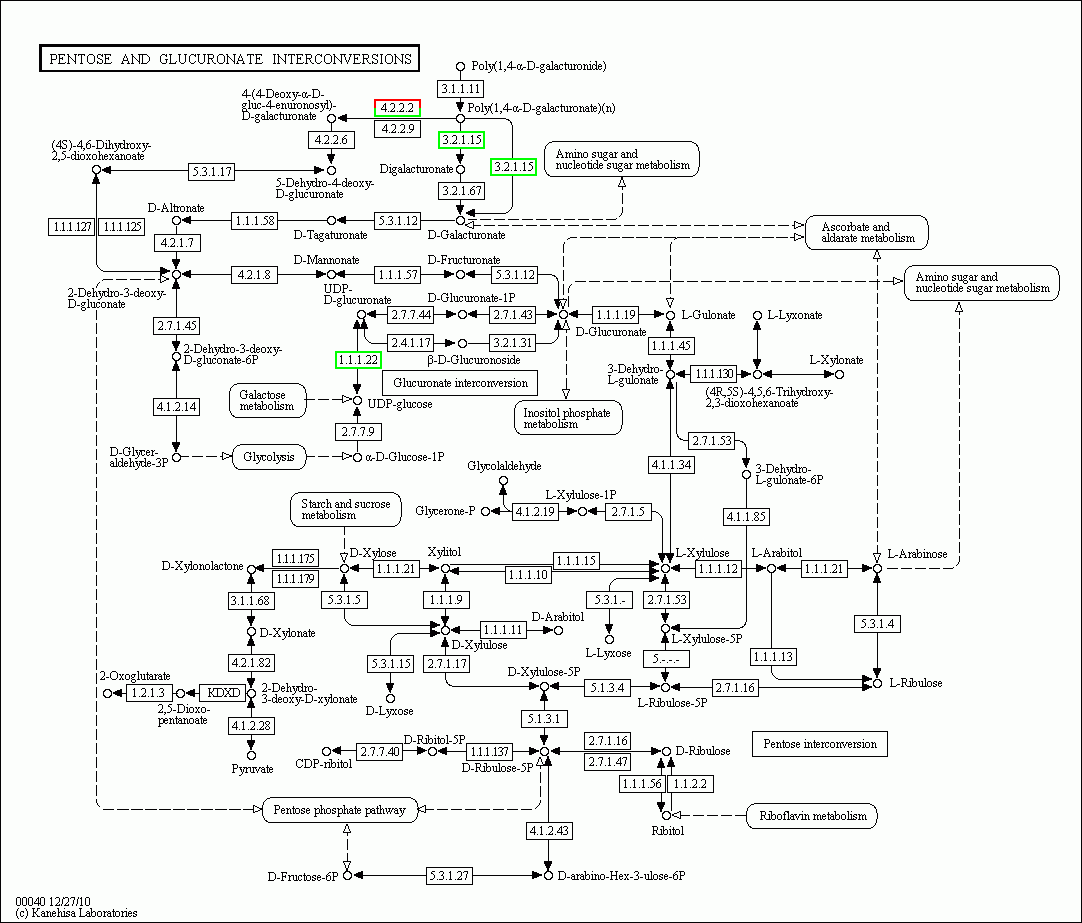


**Additional figure 2 KEGG graph of pentose and glucuronate interconversions pathway (only part of the pictures were shown )**

A, CG-vs-ETH; B, CG-vs-1-MCP. Genes with a red frame are up-regulated DEGs, while down-regulated genes are inside a green frame; genes inside a half red half green frame belong to gene families containing both up- and down-regulated DEGs. The 3.2.1.15 in the frame refers to polygalacturonase (evm.TU.supercontig_250.6, evm.TU.supercontig_92.36)
