# Supplementary material for: Isolation of ripening-related genes from ethylene/1-MCP treated papaya through RNA-seq
Source: BMC Genomics. 2017 Aug 31;18:671. doi: 10.1186/s12864-017-4072-0 (PMC5580268; doi:10.1186/s12864-017-4072-0)
Supplement: Supplementary file 3 — Selected ripening-related genes. (DOCX 23 kb) [file 12864_2017_4072_MOESM3_ESM.docx]

**Additional table 1 Selected ripening-related genes**

| **Category** | **No** | **Gene_ID** | **Definition** | **Symbol** |  | **CG_ fpkm** | **ETH_ fpkm** | **1-MCP_ fpkm** | **CG_ count** | **ETH_ count** | **1-MCP_ count** |
| --- | --- | --- | --- | --- | --- | --- | --- | --- | --- | --- | --- |
| **Cell wall-related genes** | 1 | evm.TU.supercontig_250.6 | Polygalacturonase 1 (FJ007644.1) | PG1 |  | 2554.37 | 15642.80 | 7.02 | 38771 | 115827 | 74 |
|  | 2 | evm.TU.supercontig_92.36 | Polygalacturonase 2 (GQ479794.1) | PG2 |  | 4.15 | 12.09 | 1.66 | 83 | 118 | 23 |
|  | 3 | evm.TU.supercontig_125.9 | Polygalacturonase 3 (GQ479795.1) | PG3 |  | 8.76 | 32.85 | 0.08 | 153 | 280 | 1 |
|  | 4 | evm.TU.supercontig_93.34 | Beta-galactosidase (EU650664.2) | GAL-B |  | 1796.77 | 4150.97 | 177.45 | 55521 | 62789 | 3781 |
|  | 5 | evm.TU.supercontig_5.270 | Pectate lyase | PL |  | 1.14 | 8.91 | 0.71 | 21 | 80 | 9 |
|  | 6 | evm.TU.supercontig_145.20 | Probable pectinesterase | PME |  | 2.02 | 7.29 | 0.30 | 29 | 51 | 3 |
|  | 7 | evm.TU.supercontig_1461.3 | Pectin methyl esterase | PME |  | 0.94 | 7.43 | 0.14 | 20 | 77 | 2 |
|  | 8 | evm.TU.supercontig_3.77 | Probable pectinesterase/pectinesterase inhibitor | PMIS |  | 51.28 | 4.63 | 84.91 | 1291 | 57 | 1476 |
|  | 9 | evm.TU.supercontig_80.123 | Beta glucosidase | GLU-B |  | 954.26 | 1076.07 | 366.56 | 5686 | 3095 | 1546 |
|  | 10 | evm.TU.supercontig_1.14 | Xyloglucan endotransglucosylase (AY032600.1) | XTH32 |  | 5192.30 | 8752.70 | 3204.95 | 51599 | 42280 | 22255 |
|  | 11 | evm.TU.supercontig_208.1 | Xyloglucan endotransglucosylase/ hydrolase 30 | XTH30 |  | 317.04 | 933.22 | 125.59 | 4366 | 6265 | 1202 |
|  | 12 | evm.TU.supercontig_2.231 | Endoglucanase 8-like | EGase |  | 113.73 | 355.27 | 35.80 | 2416 | 3689 | 526 |
|  | 13 | evm.TU.supercontig_106.45 | Endoxylanase (AY138968.1) | EXY1 |  | 42.35 | 895.69 | 0.24 | 1027 | 10624 | 4 |
|  | 14 | evm.TU.supercontig_106.54 | Beta-D-xylosidase 5 | XYL |  | 152.09 | 760.49 | 60.68 | 5150 | 12609 | 1416 |
|  | 15 | evm.TU.supercontig_82.65 | Sucrose synthase 4 | SUS4 |  | 79.11 | 162.30 | 81.54 | 2767 | 2780 | 1965 |
|  | 16 | evm.TU.supercontig_1178.2 | Sugar transporter 1 | STP1 |  | 5.60 | 20.41 | 3.52 | 57 | 101 | 25 |
|  | 17 | evm.TU.supercontig_40.33 | Sugar transporter 14 | STP14 |  | 74.06 | 203.34 | 39.22 | 1559 | 2092 | 571 |
|  | 18 | evm.TU.supercontig_183.26 | UDP-galactose transporter 3 | UTR3 |  | 81.17 | 195.27 | 38.90 | 980 | 1148 | 327 |
|  | 19 | evm.TU.supercontig_3.313 | Beta-fructofuranosidase | BFF |  | 6.35 | 19.24 | 0.53 | 174 | 258 | 10 |
|  | 20 | evm.TU.supercontig_1.419 | Expansin A | EXPA |  | 2997.11 | 6748.84 | 1057.26 | 25421 | 27775 | 6287 |
| **Chlorophyll and carotenoid metabolism-related genes** | 1 | evm.TU.supercontig_5.220 | Ferretin 1 | FER1 |  | 66.07 | 27.70 | 140.30 | 570 | 116 | 848 |
|  | 2 | evm.TU.supercontig_43.43 | Hydroxymethylbilane synthase | HEMC |  | 4.77 | 2.26 | 4.55 | 91 | 21 | 60 |
|  | 3 | evm.TU.supercontig_130.4 | Protoporphyrinogen oxidase | HEMG |  | 2.72 | 0.90 | 5.20 | 56 | 9 | 74 |
|  | 4 | evm.TU.supercontig_92.51 | Magnesium-chelatase subunit chlH | ABAR |  | 0.21 | 0.00 | 0.35 | 13 | 0 | 15 |
|  | 5 | evm.TU.supercontig_7.16 | Magnesium-chelatase subunit ChlI | CH-42 |  | 0.76 | 0.00 | 1.29 | 11 | 0 | 13 |
|  | 6 | evm.TU.supercontig_13.164 | Magnesium-protoporphyrin IX methyltransferase | CHLM |  | 3.59 | 1.41 | 7.00 | 42 | 8 | 57 |
|  | 7 | evm.TU.supercontig_80.29 | Protochlorophyllide oxidoreductase A | PORA |  | 0.87 | 0.00 | 0.92 | 15 | 0 | 11 |
|  | 8 | evm.TU.supercontig_23.100 | NAD(P)-binding rossmann-fold superfamily protein | NYC1 |  | 66.24 | 29.69 | 86.92 | 1465 | 321 | 1329 |
|  | 9 | evm.TU.supercontig_44.117 | Chlorophyllase 2 | CLH2 |  | 47.15 | 19.27 | 48.92 | 528 | 105 | 382 |
|  | 10 | evm.TU.supercontig_47.4 | Pheophorbide a oxygenase, chloroplastic-like | ACD1 |  | 34.08 | 10.77 | 39.13 | 648 | 100 | 515 |
|  | 11 | evm.TU.contig_43305 | Phytoene synthase (DQ666828.1) | PSY |  | 278.16 | 360.59 | 196.22 | 1230 | 765 | 621 |
|  | 12 | evm.TU.supercontig_157.3 | Phytoene desaturase 3 (DQ666830.2) | PDS |  | 233.08 | 216.05 | 202.65 | 4092 | 1852 | 2465 |
|  | 13 | evm.TU.supercontig_20.108 | Phytoene desaturase 3 | PDS |  | 7.04 | 0.85 | 3.28 | 152 | 9 | 49 |
|  | 14 | evm.TU.supercontig_117.67 | Zeta-carotene desaturase (DQ666829.2) | ZDS |  | 561.68 | 591.61 | 509.75 | 13295 | 6848 | 8336 |
|  | 15 | evm.TU.supercontig_195.16 | Chromoplast-specific lycopene beta-cyclase (FJ839871.1) | CYC-B |  | 7.05 | 8.21 | 11.08 | 123 | 70 | 134 |
|  | 16 | evm.TU.supercontig_5.198 | Lycopene beta-cyclase (FJ599643.1) | LCY-B |  | 16.4 | 6.81 | 26.05 | 85 | 17 | 96 |
|  | 17 | evm.TU.supercontig_132.5 | Lycopene beta/epsilon cyclase protein isoform 2 | LCY-B/E |  | 3.6 | 1.02 | 3.22 | 58 | 8 | 36 |
|  | 18 | evm.TU.supercontig_107.106 | Beta-carotene hydroxylase (HQ998850.1) | CHY-B |  | 1324.14 | 2596.23 | 646.38 | 12709 | 12108 | 4338 |
| **Proteinases and their inhibitors** | 1 | evm.TU.supercontig_12.94 | Subtilisin-like serine endopeptidase family protein | AIR3 |  | 153.14 | 1456.31 | 10.49 | 4851 | 22584 | 229 |
|  | 2 | evm.TU.supercontig_4.28 | Cysteine proteinases superfamily protein | CPSP |  | 577.67 | 3407.14 | 260.70 | 7787 | 22386 | 2443 |
|  | 3 | evm.TU.supercontig_228.9 | Cysteine proteinase inhibitor | CYS |  | 676.26 | 365.06 | 734.03 | 5014 | 1311 | 3828 |
|  | 4 | evm.TU.contig_38010 | Serine protease inhibitor family protein | SERPIN |  | 119.41 | 48.73 | 103.75 | 1065 | 211 | 648 |
| **Plant hormone signal transduction pathway genes** | 1 | evm.TU.supercontig_25.85 | ABA insensitive protein | HAB1 |  | 68.07 | 22.40 | 70.57 | 1096 | 176 | 788 |
|  | 2 | evm.TU.supercontig_35.27 | Cytokinin response regulator 5 | ARR5 |  | 24.92 | 40.23 | 11.79 | 140 | 109 | 47 |
|  | 3 | evm.TU.supercontig_292.1 | Auxin-responsive GH3 family protein | GH3.1 |  | 2.52 | 17.92 | 0.58 | 63 | 219 | 10 |
|  | 4 | evm.TU.supercontig_151.32 | Ethylene receptor (AF311942.1) | ERS |  | 313.33 | 491.22 | 56.01 | 8405 | 6447 | 1037 |
|  | 5 | evm.TU.supercontig_750.1 | EIN3 binding F-box 2 (KF709951.1) | EBF2 |  | 146.25 | 159.79 | 366.00 | 4001 | 2139 | 6910 |
|  | 6 | evm.TU.supercontig_2388.2 | Ethylene-insensitive 4-like protein | EIN4 |  | 61.08 | 70.53 | 21.25 | 1997 | 1129 | 479 |
| **Transcription factors** | 1 | evm.TU.supercontig_1.48 | NAC domain protein | NAC |  | 17.67 | 3.55 | 22.37 | 83 | 8 | 75 |
|  | 2 | evm.TU.supercontig_165.12 | NAC domain protein | NAC |  | 12.58 | 24.53 | 1.46 | 136 | 129 | 11 |
|  | 3 | evm.TU.supercontig_18.82 | WRKY DNA-binding protein | WRKY |  | 13.05 | 2.32 | 33.97 | 150 | 13 | 272 |
|  | 4 | evm.TU.supercontig_19.44 | WRKY DNA-binding protein | WRKY |  | 38.09 | 12.40 | 177.08 | 373 | 59 | 1212 |
| **Senescence-associated gene** | 1 | evm.TU.supercontig_99.60 | Senescence-associated gene | SAG |  | 279.89 | 1111.56 | 144.87 | 5078 | 953 | 2632 |
