# Supplementary material for: Isolation of ripening-related genes from ethylene/1-MCP treated papaya through RNA-seq
Source: BMC Genomics. 2017 Aug 31;18:671. doi: 10.1186/s12864-017-4072-0 (PMC5580268; doi:10.1186/s12864-017-4072-0)
Supplement: Supplementary file 4 — KEGG graph of porphyrin and chlorophyll metabolism pathway (CG-vs-ETH). 1.16.3.1 indicates ferretin 1 (evm.TU.supercontig_5.220); 2.5.1.61 indicates hydroxymethylbilane synthase (evm.TU.supercontig_43.43); 1.3.3.4 indicates protoporphyrinogen oxidase (evm.TU.supercontig_130.4); 6.6.1.1 indicates magnesium-chelatase subunit ChlI (evm.TU.supercontig_7.16) and magnesium-chelatase subunit chlH (evm.TU.supercontig_92.51); 2.1.1.11 indicates magnesium-protoporphyrin IX methyltransferase (evm.TU.supercontig_13.164); 1.3.1.33 indicates protochlorophyllide oxidoreductase A (evm.TU.supercontig_80.29); 1.1.1294 indicates NAD (P)-binding rossmann-fold superfamily protein (evm.TU.supercontig_23.100); 3.1.1.14 indicates chlorophyllase 2 (evm.TU.supercontig_44.117); 1.14.1220 indicates pheophorbide a oxygenase, chloroplastic-like (evm.TU.supercontig_47.4). (DOCX 85 kb) [file 12864_2017_4072_MOESM4_ESM.docx]

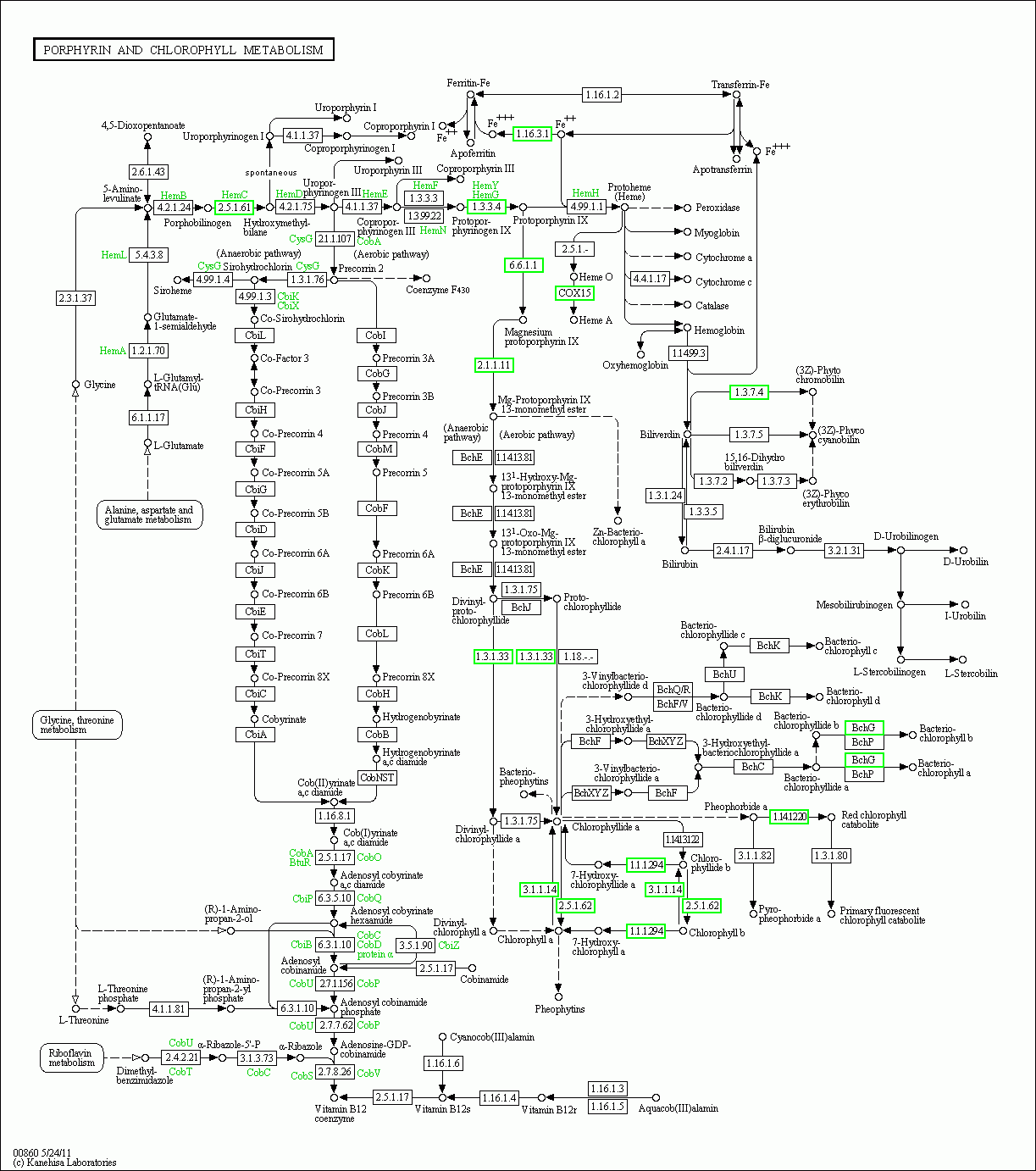


**Additional figure 3 KEGG graph of porphyrin and chlorophyll metabolism pathway (**CG-vs-ETH**)**

1.16.3.1 indicates ferretin 1 ( evm.TU.supercontig_5.220 ); 2.5.1.61 indicates hydroxymethylbilane synthase (evm.TU.supercontig_43.43); 1.3.3.4 indicates protoporphyrinogen oxidase (evm.TU.supercontig_130.4); 6.6.1.1 indicates magnesium-chelatase subunit ChlI (evm.TU.supercontig_7.16) and magnesium-chelatase subunit chlH (evm.TU.supercontig_92.51); 2.1.1.11 indicates magnesium-protoporphyrin IX methyltransferase (evm.TU.supercontig_13.164); 1.3.1.33 indicates protochlorophyllide oxidoreductase A (evm.TU.supercontig_80.29); 1.1.1294 indicates NAD(P)-binding rossmann-fold superfamily protein (evm.TU.supercontig_23.100); 3.1.1.14 indicates chlorophyllase 2 (evm.TU.supercontig_44.117); 1.14.1220 indicates pheophorbide a oxygenase, chloroplastic-like (evm.TU.supercontig_47.4)
