# Supplementary material for: Isolation of ripening-related genes from ethylene/1-MCP treated papaya through RNA-seq
Source: BMC Genomics. 2017 Aug 31;18:671. doi: 10.1186/s12864-017-4072-0 (PMC5580268; doi:10.1186/s12864-017-4072-0)
Supplement: Supplementary file 5 — KEGG graph of carotenoid biosynthesis pathway. A, CG-vs-ETH; B, CG-vs-1-MCP. The CruA indicates lycopene beta/epsilon cyclase protein (evm.TU.supercontig_132.5); The CrtR and CrtZ indicate beta-carotene hydroxylase (evm.TU.supercontig_107.106). (DOCX 57 kb) [file 12864_2017_4072_MOESM5_ESM.docx]

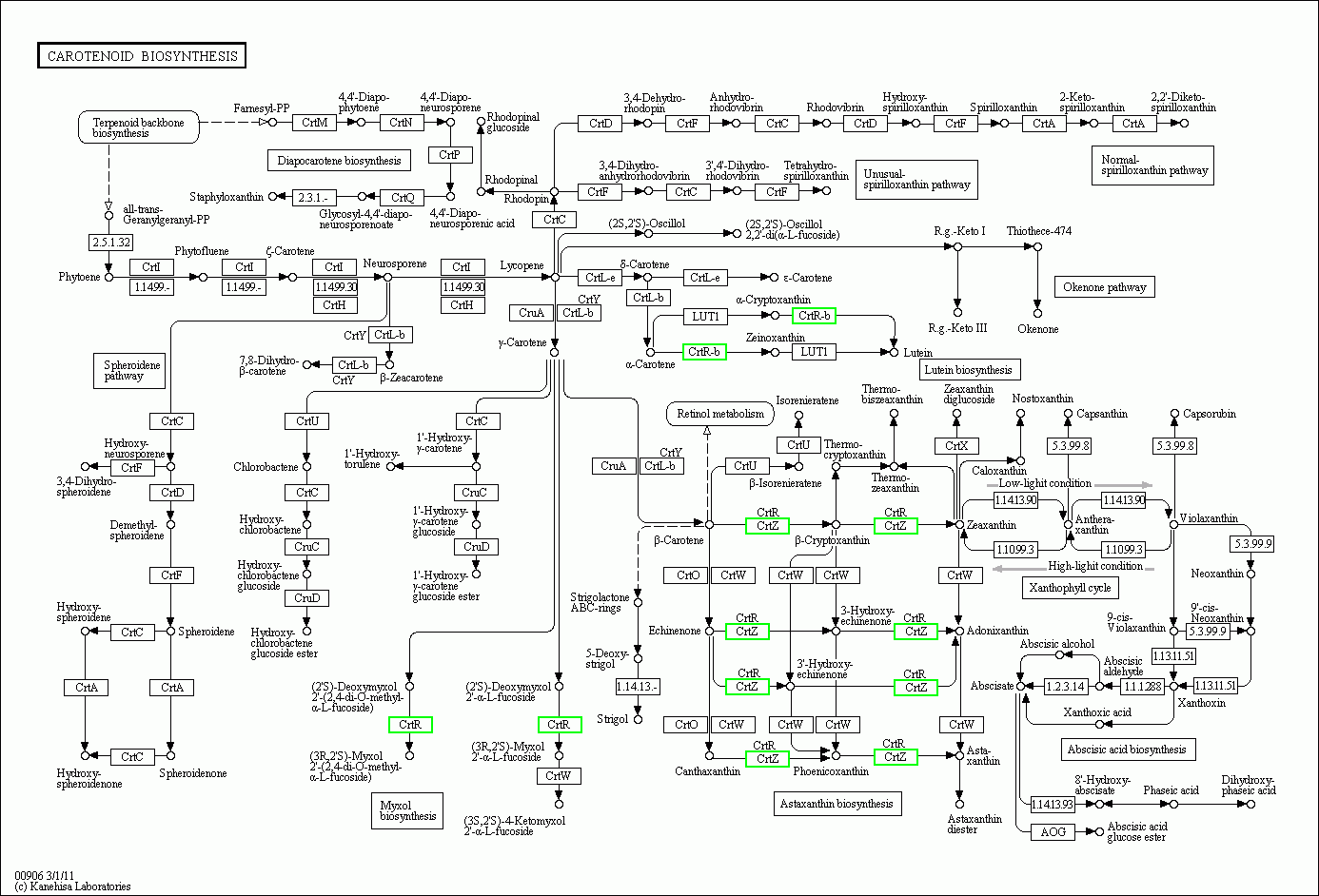

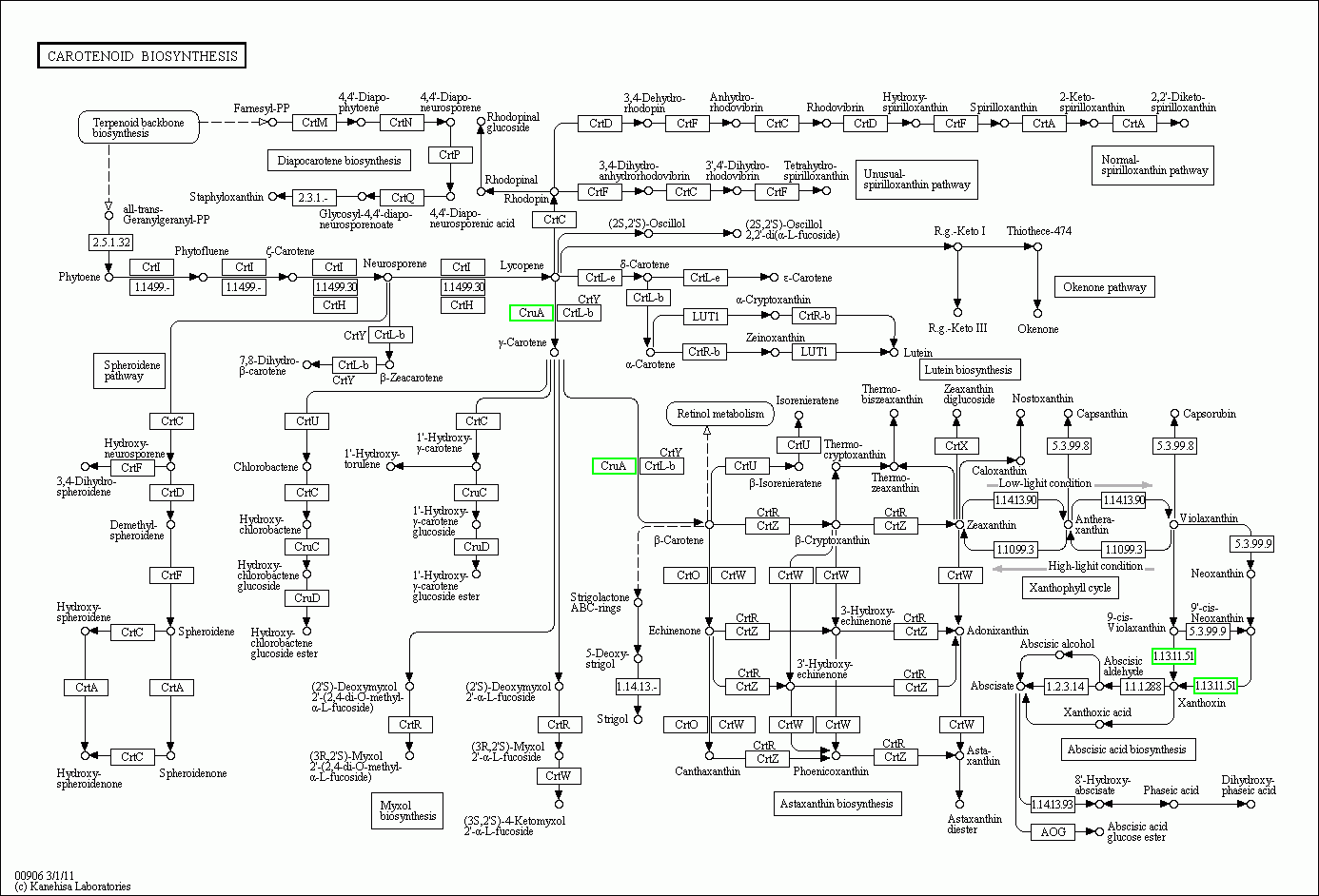


B

**A**

B

**Additional figure 4 KEGG graph of carotenoid biosynthesis pathway**

A, CG-vs-ETH; B, CG-vs-1-MCP.

The CruA indicates lycopene beta/epsilon cyclase protein ( evm.TU.supercontig_132.5); The CrtR and CrtZ indicate beta-carotene hydroxylase (evm.TU.supercontig_107.106)
