# Supplementary material for: Isolation of ripening-related genes from ethylene/1-MCP treated papaya through RNA-seq
Source: BMC Genomics. 2017 Aug 31;18:671. doi: 10.1186/s12864-017-4072-0 (PMC5580268; doi:10.1186/s12864-017-4072-0)
Supplement: Supplementary file 6 — General pathway of carotenoid metabolism. (DOCX 26 kb) [file 12864_2017_4072_MOESM6_ESM.docx]

GGPP

↓PSY

Phytoene

↓PDS

ζ-Cartotene

↓ZDS

Lycopene

LCY-ε↙ ↘LCY-β

δ-Cartotene γ-Cartotene

LCY-β ↓ ↓LCY-β

α-Cartotene β-Cartotene

CHY-β / OHase-ε ↓ ↓CHY-β

Lutein β-cryptoxanthin

↓CHY-β

Zeaxanthin

**β-branch**

**α-branch**

**Additional figure 5 General pathway of carotenoid metabolism**
